# The Antitoxin Treatment of Distemper or Shipping Fever in Horses

**Published:** 1900-03

**Authors:** George S. Fuller

**Affiliations:** Philadelphia, Pa.


### THE ANTITOXIN TREATMENT OF DISTEMPER OR SHIPPING FEVER IN HORSES.

By GEORGE S. FULLER, V.S.,
PHILADELPHIA, PA.

AFTER more than a year's treatment of large stables of horses, subjected to the conditions which almost invariably induce influenza or shipping fever, I am convinced that distemper antitoxin is a valuable preventive of the disease.

When used in the earlier stages of influenza the cases can sometimes be aborted with the result of a normal temperature, disappearance of congestive and catarrhal symptoms, and return of appetite within forty-eight hours. In other cases the disease will run its regular course with the usual symptoms, except that the heart always behaves much better and the appetite and digestion remain good, which of course leaves the patient fit for work much sooner than if there had been no serum used.

I have used considerable of it on green western horses in the stables of Messrs. L—— Bros., and have seldom had an animal that was inoculated on his arrival lose any time on account of distemper. I allowed one lot of five green horses, during the pleasant weather of early winter, to go without immunizing, and within the first four weeks had every one sick. Since then I have immunized three out of a lot of five that came in together, leaving two of the older ones that looked somewhat second-handed without the antitoxin. These two are, at this writing, sick, and the three younger ones are perfectly well, all having received the same feed, care, and work.

As to the method of using, I have, after considerable experimenting, arrived at the following conclusion: When an animal, green or acclimated, is well one dose of 20 c.c. is sufficient to give him immunity from influenza for as long a time as actual occurrence of the disease would have afforded. If the disease has already been ushered in and there are present some congestive and catarrhal symptoms with moderate elevation of temperature, I administer 20 c.c., followed next day by a second dose of 10 c.c., when, if symptoms are all abating, I look for recovery the next day. If, on the other hand, I find no improvement on the second examination, I put the patient on the treatment indicated by his condition, confident that the administration of the first 20 c.c. will modify the usual course of the disease as stated above. I have tried pushing the antitoxin[1] in large and repeated doses, but found no benefit from it that would not accrue from the first injection of 20 c.c.

[1] The antitoxin employed in all my cases was that furnished by the H. K. Mulford Co.

